# Leukocyte Segmentation Method Based on Adaptive Retinex Correction and U-Net

**DOI:** 10.1155/2022/9951582

**Published:** 2022-07-04

**Authors:** Wei Chen, Mengjing Zhu

**Affiliations:** School of Communication and Information Engineering, Xi'an University of Science and Technology, Shaanxi 710054, China

## Abstract

To address the issues of uneven illumination and inconspicuous leukocyte properties in the gathered cell pictures, a leukocyte segmentation method based on adaptive retinex correction and U-net was proposed. The procedure begins by processing a peripheral blood image to clearly distinguish leukocytes from other components in the image. The adaptive retinex correction, which is based on multiscale retinex with colour replication (MSRCR), redefines the colour recovery function by introducing Michelson contrast. Then, the image is trained with the U-net convolutional neural network, and the leukocyte segmentation is completed. The innovation is in the manner of processing peripheral blood images, which improves the accuracy of leukocyte segmentation. This study conducts experimental evaluations on the Cellavision, BCCD, and LISC datasets. The experimental results show that the method in this study is better than the current best method, and the segmentation accuracy rate reaches 98.87%.

## 1. Introduction

Leukocytes are an important part of the human immune system. Neutrophils, basophils, eosinophils, monocytes, and lymphocytes make up the majority of leukocytes [1]. Because the number and proportion of different types of leukocytes correspond to different diseases, leukocyte detection accuracy is critical for disease auxiliary diagnosis [2].

Manual microscopy is used in the most traditional leukocyte detection procedures. Manual microscopic examination is the “gold standard” of clinical examination. It requires two well-trained and experienced laboratory physicians to observe the morphology and count of leukocytes under the microscope and finally determine the test results. This strategy is not only inefficient and time-consuming but also has a high level of subjectivity [3].

Leukocytes are now segmented and identified using traditional image processing. These include watershed algorithms [4], edge detection [5], active contour models [6], and adaptive threshold segmentation [7]. However, the traditional method can only conduct single-label classification and cannot perform multilabel classification, and it still has issues such as incomplete cell segmentation and low accuracy. Edge detection is only capable of generating edge points, not completing image segmentation, and must be processed further after acquiring edge point information [5]. The active contour model relies on the initial contour being chosen correctly; otherwise, a suitable segmentation result cannot be obtained if the starting contour is incorrect [6]. In adaptive threshold segmentation, choosing an adequate threshold is critical because it is susceptible to noise and has low resilience [7]. As a result, the traditional method is inadequate for today's practical use.

With improvements in computer performance and the increase in data volume in recent years, deep learning has also been widely used in leukocyte detection [8]. Ma et al. [9] proposed an improved residual neural network (ResNet) to classify leukocytes, with an accuracy rate of 92%. Kutlu et al. [10] proposed a regional convolutional neural network (R-CNN) based on CNN to identify white blood cells. Lymphocytes, monocytes, basophils, eosinophils, and neutrophils had accuracy rates of 99.52%, 98.40%, 98.48%, 96.16%, and 95.04%, respectively. Geng et al. [11] integrated the attention mechanism module on the Mask R-CNN structure, and the average segmentation accuracy MIoU achieved 89.30%.

The current study discovered that the public dataset has few leukocyte data samples, that many studies require data extension, and that only a single leukocyte segmentation can be done in an image. This study proposes a leukocyte segmentation approach based on adaptive retinex correction and U-net to address the aforementioned issues. To improve the robustness of training, an adaptive retinex method is used to preprocess the blood cell image, strengthen the different characteristics of individual leukocytes, and eliminate the problem of uneven illumination during acquisition. Then, U-net is used to train and predict the processed dataset; U-net can train with fewer datasets, which solves the problem of fewer leukocyte data samples.

## 2. Methods

**Figure 1:**
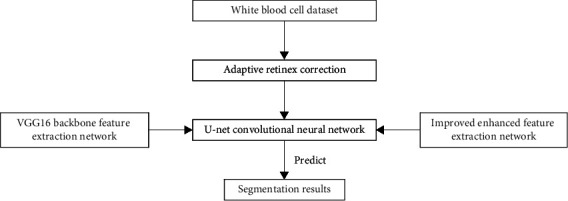
Schematic diagram of the leukocyte segmentation model.

The method presented in this study is separated into two parts: image preprocessing using adaptive retinex correction and image semantic segmentation using a U-net network. Among them, the U-net network completes the segmentation of the pixels in the blood cell image. Pure semantic segmentation, however, is unable to correctly segment leukocytes due to the uneven backdrop and a significant number of other cells in the blood cell image. As a result, in this study, we used the adaptive retinex method to preprocess blood cells, reducing uneven illumination during collection and improving leukocyte properties. For semantic segmentation, the generated images reveal precise leukocyte positions.  Figure 1 shows a schematic diagram of the method.

### 2.1. Adaptive Retinex Correction

**Figure 2:**
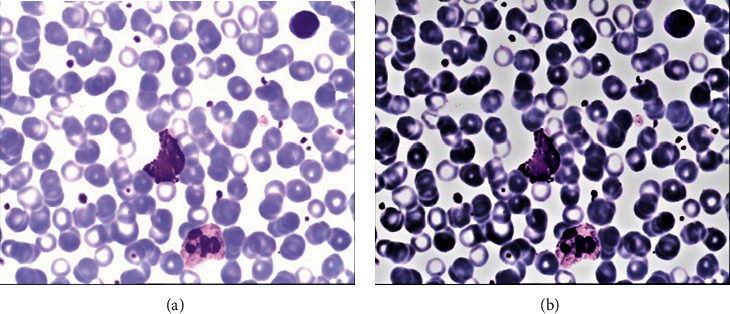
Result plot processed with MSRCR. (a) Original image. (b) After MSRCR processing.

Single-scale retinex (SSR) and multiscale retinex (MSR) are two of the most well-known algorithms in retinex theory. Multiscale retinex with colour restoration (MSRCR) [12] is offered as a solution to the colour distortion problem. It did not produce satisfactory results when used in the processing of blood cell images. The processing result is shown in Figure 2. In the processed image, the hue of leukocytes and other blood cells is more similar, causing some interference in the subsequent training. Therefore, this study presents an adaptive retinex correction to process the image, which may better separate the background from the leukocytes while also clarifying the leukocyte properties.

The fundamental assumption of retinex theory is that the original image *S* is the product of the lighting image *L* and the reflectance image *R*, which may be stated as follows:

(1) $$\begin{array}{c} S\left(x,y\right)=R\left(x,y\right)\cdot L\left(x,y\right). \end{array}$$

In processing, it is usually transferred to the logarithmic domain, that is, *s* = log(*S*), *l* = log(*L*), and *r* = log(*R*), to convert the product relationship into a sum relationship:

(2) $$\begin{array}{c} \log\left(S\right)=\log\left(R\cdot L\right), \end{array}$$

(3) $$\begin{array}{c} \log S=\log R+\log L, \end{array}$$

(4) $$\begin{array}{c} s=r+1. \end{array}$$

Retinex theory works by estimating the illumination *L* from the original image *S*, deconstructing the reflection component *R*, decreasing the influence of uneven illumination, and boosting the visual effect of the image, which is similar to how the human visual system works. That is,

(5) $$\begin{array}{c} l=f\left(s\right), \end{array}$$

(6) $$\begin{array}{c} r=s-f\left(s\right). \end{array}$$

Following that, the SSR implementation steps are as follows:

(Step 1) Divide the image into three channels *R*, *G*, and *B*, and apply the logarithmic transformations described in Formulas (2) and (3).
(Step 2) Create a Gaussian surround function, convolve the grayscale images of each channel with the Gaussian surround function, and obtain the illumination estimation components for the three channels.
(Step 3) In the logarithmic domain, execute a difference operation on the original image and the Gaussian blurred image to retrieve the reflection component.
(Step 4) Linearly stretch or exponentially transform the obtained reflection component's result into the data type of image output.
(Step 5) To create an SSR-enhanced image, the three-channel reflection component images are combined into one image.

**Figure 3:**
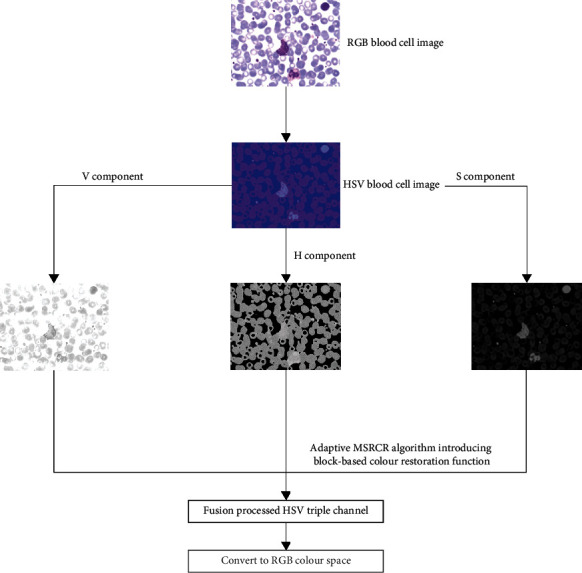
Adaptive retinex correction process.

The MSR selects three scale parameters in Step 2 of the SSR to form three Gaussian surround functions and then performs convolution and weighted averaging to obtain the illumination estimation components of each channel to effectively maintain details and colour information. MSRCR is based on MSR. In Step 4, the colour recovery function is used to multiply the MSR enhancement function of each channel to obtain the image enhancement reflection component of the three channels to reduce the colour shift. The adaptive retinex algorithm proposed in this study converts the image in RGB colour space to HSV colour space on the basis of MSRCR [13]. To provide colour correction for the *H* and *S* components, a block-based colour recovery mechanism is introduced. The *H*- and *S*-component images are first separated into n small blocks of *k* × *k* [14], and then, Michelson contrast is applied to each small block to select the appropriate *β*.  Figure 3 depicts the overall algorithm processing procedure.

The MSRCR model is as follows:

(7) $$\begin{array}{c} F_{\text{MSRCR}}\left(x,y\right)=c_{i}\left(x,y\right)F_{\text{MSR}}\left(x,y\right), \end{array}$$

where *c*_*i*_(*x*, *y*) represents the colour recovery function, and the formula satisfies

(8) $$\begin{array}{c} c_{i}\left(x,y\right)=\alpha \cdot \log\left[\beta \cdot \frac{S_{i}\left(x,y\right)}{\sum_{i}S_{i}\left(x,y\right)}\right], \end{array}$$

where *i* ∈ {*R*, *G*, *B*}, *α*, and *β* are the gain factor offsets that affect the colour recovery of the image, and they are all constants. Generally, the value of *α* is 46, and the value of *β* is 125.

*β* is employed as a constant, and the colour recovery function and each pixel in the MSR-enhanced image must be multiplied according to Formula (7). As a result, MSRCR is used to process the Wright-stained blood cell image to ensure that the pixel point ratio between each channel is consistent with that of the original image, which will make the overall image tend to the same tone. In the blood cell image after Wright's staining, the colour of leukocytes will be darker than other cells or impurities, and the pixel value of the background is the lowest, so *β* should be changed according to different ranges of pixel values.

**Figure 4:**
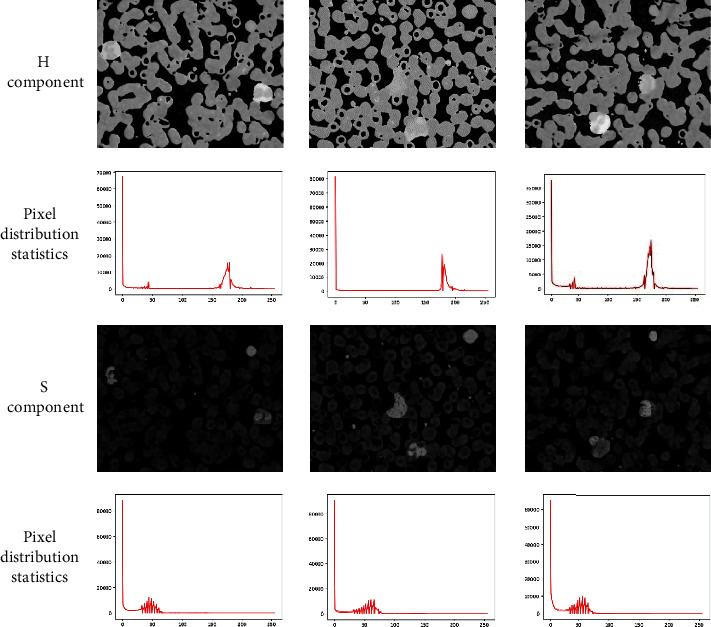
*H* and *S* components and their pixel distribution statistics.

First, the pixel distribution of the *H* and *S* components of the blood cell image is counted, and the relationship between white blood cells and other cells and the background is observed. As shown in Figure 4, the *H* component distinguishes between the background and the cells; therefore, the background is handled there to solve the problem of a too bright or too dark background. Background, other cells and impurities, and leukocytes are separated in the *S* component, so they are processed separately in the *S* component to increase the detailed features of leukocytes, and the three components are normalized independently at the same time. The MSRCR is adjusted according to the relationship between pixel values and pixels in the block-based colour recovery function developed in this study, and Michelson contrast is introduced to distinguish leukocytes from other cells and impurities.

The Michelson contrast can be defined as follows:

(9) $$\begin{array}{c} C_{M}=\frac{I_{\max}-I_{\min}}{I_{\max}+I_{\min}}, \end{array}$$

where *I*_max_ and *I*_min_ represent the brightest and darkest brightnesses, respectively.

Taking *γ* as a constant, *β* is defined as

(10) $$\begin{array}{c} \beta =\gamma \cdot \exp\left(C_{M}\right). \end{array}$$

Then, the *c*_*i*_(*x*, *y*) colour recovery function satisfies

(11) $$\begin{array}{c} c_{i}\left(x,y\right)=\alpha \cdot \log\left[\gamma \cdot \exp\left(C_{M}\right)\cdot \frac{S_{i}\left(x,y\right)}{\sum_{i}S_{i}\left(x,y\right)}\right]=\alpha \cdot \log\left[\gamma \cdot \exp\left(\frac{I_{\max}-I_{\min}}{I_{\max}+I_{\min}}\right)\cdot \frac{S_{i}\left(x,y\right)}{\sum_{i}S_{i}\left(x,y\right)}\right]. \end{array}$$

For each *k* × *k* patch, in the *H* component, *I*_max_ represents all cells and impurities in the blood cell image, and *I*_min_ represents the background in the blood cell image. When *C*_*M*_ increases gradually, it means that the contrast between the cells and the background in the image is large; then the value of *β* increases with the increase of contrast, which can suppress the influence of uneven brightness caused by nonuniform illumination; as *C*_*M*_ gradually decreases, it may be under the same black background or gray all cells and impurities in the image; the value of *β* tends to be constant, ensuring that leukocytes and other impurities are not overly similar. In the *S* component, *I*_max_ represents the leukocytes in the blood cell image, and *I*_min_ represents the background in the blood cell image. The value of *β* varies with *C*_*M*_, refining the leukocyte characteristics while increasing the contrast between leukocytes and other cells and impurities.

If the brightest and darkest brightnesses are the same, that is, *I*_max_ = *I*_min_, then *C*_*M*_ = 0, and *β* = *γ* · exp(*C*_*M*_) = *γ*, the colour recovery function is simplified to

(12) $$\begin{array}{c} c_{i}\left(x,y\right)=\alpha \cdot \log\left[\gamma \cdot \frac{S_{i}\left(x,y\right)}{\sum_{i}S_{i}\left(x,y\right)}\right]. \end{array}$$

This is the same as the original colour recovery function, but the colour recovery function designed in this study has a better effect when processing blood cell images.

### 2.2. U-Net Convolutional Neural Network

**Figure 5:**
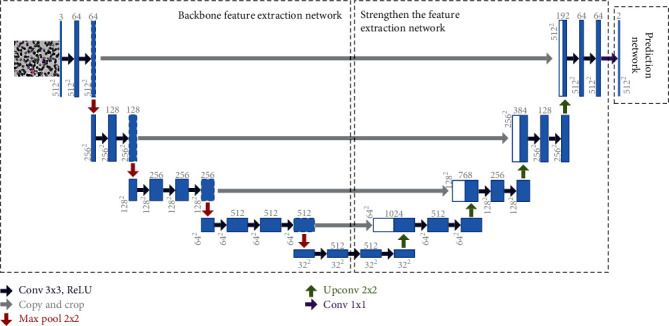
U-net semantic segmentation model in this study.

As shown in Figure 5, the U-net semantic segmentation model in this study can be divided into the following three parts:

The backbone feature extraction network is the initial part, and the VGG16 network is employed in this study. Using the backbone feature extraction component, five preliminary effective feature layers may be created, and these five effective feature layers are used for feature fusion in the second part.

The strengthening of the feature extraction network is the second step. This part performs upsampling and feature fusion on the five preliminary effective feature layers acquired in the first phase, resulting in a final effective feature layer that incorporates all features. Upsampling is directly doubled in this part, followed by feature fusion to optimize the height and breadth of the augmented feature layer to match the backbone feature layer.

The prediction network is the third part, and it employs the last effective feature layer to categorize each feature point, which is equivalent to classifying each pixel point to obtain a segmentation result.

## 3. Experiment

### 3.1. Lab Environment

The experiments in this study are based on the deep learning framework TensorFlow, and the experimental environment is Python3.6. The processor is i7-9700f, the memory is 8G, the graphics card is GTX1660, and the operating system is Windows 10.

### 3.2. Model Training

The dataset used in this study is the LISC dataset. There are 250 image samples in all. The number is sufficient, and the cell image condition is somewhat complex; therefore, the training set is essentially met. Eighty of them were selected. First, the methods proposed in this study were applied to these 80 datasets. Then, the sample dataset is divided into 40 training and testing sets. Finally, the model is trained with 40 training sets using U-net. In the experiment, the pretrained weights are the initialization weights of the VGG16 network. All the training data are normalized to 512 × 512 × 3, and then training is started from the 0th generation. The model is first frozen for 50 generations and then trained for 50 generations after thawing. Two data samples were captured each time. The initial learning rate is set to 1 × 10^−4^.

### 3.3. Dataset

This study conducts comparison experiments using different datasets to verify the effectiveness of the proposed method. Cellavision, BCCD, and LISC datasets are used in this study. Among them, in addition to the BCCD dataset, other datasets contain five types of leukocytes, namely, neutrophils, lymphocytes, monocytes, eosinophils, and basophils. The BCCD dataset is devoid of basophils. The LISC dataset [15] was obtained from the Imam Khomein Hospital's Center for Heamatology-Oncology and BMT Research in Tehran, Iran, and comprises 250 cell images at a resolution of 720 × 576 pixels. The Cellavision dataset [16] contains 100 JPG-formatted colour photos at a resolution of 300 × 300 pixels. The BCCD dataset consists of 364 blood cell images of 416 × 416 pixels.

### 3.4. Evaluation Metrics

**Figure 6:**
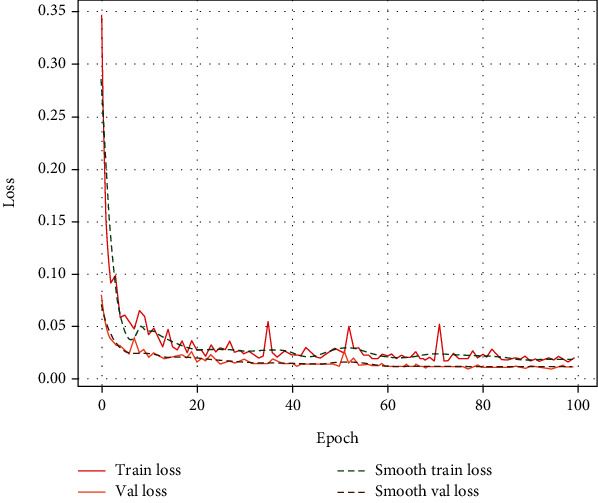
Loss curve.

Train_loss and val_loss are presented in this study to check whether the parameter choices of the U-net network model training are adequate. Train_loss is the loss on the training data, which measures the fitting ability of the model on the training set. Val_loss is the loss on the validation set, which measures the fitting ability on the test set, which can also be said to have the generalization ability. As shown in Figure 6, both train_loss and val_loss decrease continuously, indicating that the network training state is normal.

To evaluate the image segmentation performance, four parameters, the Dice coefficient, mean intersection over union (MioU), mean pixel accuracy (MPA), and accuracy, were selected. Suppose that set *X* represents the set of predicted value pixels obtained by the algorithm model, set *Y* represents the set of artificially labelled ground-truth pixels, and *k* represents the total number of categories. Then, each parameter is defined as follows.

#### 3.4.1. Dice Coefficient

A set similarity measure function calculates the similarity of two samples. The value range is [0, 1]; the closer the value to 1, the better the model effect. The definition is shown in

(13) $$\begin{array}{c} \text{Dice}=\frac{2\left|X\cap Y\right|}{\left|X\right|+\left|Y\right|}. \end{array}$$

#### 3.4.2. MioU

It is a standard measure for semantic segmentation, calculating the ratio of the intersection and union of two sets of true and predicted values. The definition is shown in

(14) $$\begin{array}{c} \text{MIoU}=\frac{1}{k}\overset{k}{\underset{i=1}{\sum }}\frac{X\cap Y}{X\cup Y}. \end{array}$$

#### 3.4.3. MPA

It is the average of the sum of pixel accuracies across all classes. The definition is shown in

(15) $$\begin{array}{c} \text{MPA}=\frac{1}{k+1}\overset{k}{\underset{i=0}{\sum }}\frac{X\cap Y}{\sum_{j=0}^{k}\left(Y-X\cap Y\right)}. \end{array}$$

#### 3.4.4. Accuracy

It shows the accuracy of all predicted values compared to the true values. The IoU value is used as a comparison parameter. Generally, when the IoU of a prediction result for a specific category is more than 0.5, the category of the prediction result is considered accurate. However, to obtain more accurate results, the critical value of IoU is set as 0.8. The definition is shown in

(16) $$\begin{array}{c} \text{Accuracy}=\frac{\text{number of IoU}>0.8}{\text{total number of forecast samples}}. \end{array}$$

## 4. Results

### 4.1. Comparison Results of the LISC Dataset

**Figure 7:**
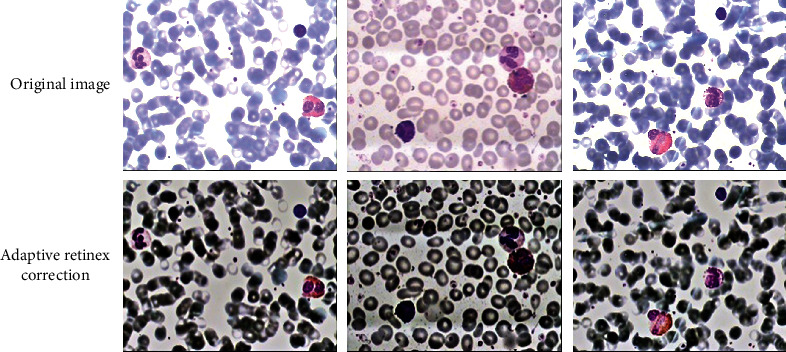
Adaptive retinex correction of blood cell image results.

Adaptive retinex corrected blood cell images and U-net-based leukocyte segmentation and classification are the two primary sections of this study.  Figure 7 shows the image processing results of adaptive retinex corrected blood cells. Three images with different background brightnesses of the original image were selected for comparison, and good results were obtained from the processing results. The backdrops tend to be uniformly bright, which eliminates the problem of backgrounds that are either too bright or too dark. Other cells and impurities distinguish leukocytes clearly, giving the groundwork for training. The features of leukocytes are also more distinct than in the original image.

**Figure 8:**
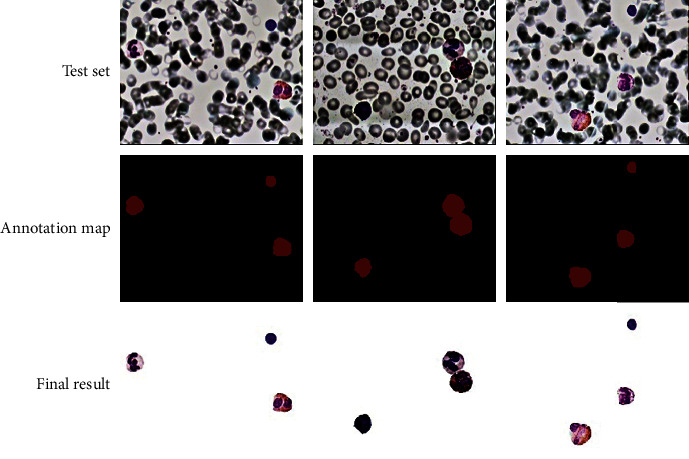
Segmentation result.

The segmentation results of any three blood cell images in the 40 test sets are shown in Figure 8. The images in the test set are photos of leukocytes with noticeable characteristics after the adaptive retinex in this study has corrected the original blood cell images. The result of manual labelling is the annotation map, which serves as a comparison to the segmentation result. The final segmented image is obtained by removing the background and leaving only the leukocytes on the original image for the predicted segmentation result. The accuracy value ultimately reached 98.87%.

The training of convolutional neural networks is carried out using the original data samples and the preprocessed data samples to validate that the method suggested in this study has a better effect.  Table 1 shows the before and after results for MioU, MPA, and Accuracy. The findings demonstrate that the preprocessing method utilized in this study has improved significantly. Not only is the number of data samples used cut in half, but MioU is improved by 22.09%, MPA by 15.53%, and accuracy by 15.24%.

### 4.2. Comparison Results of Different Segmentation Methods on Leukocytes

**Figure 9:**
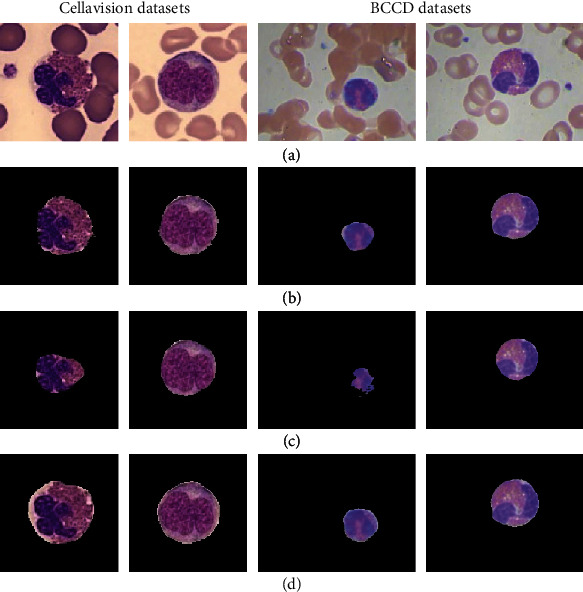
Segmentation results of different CNN methods in different datasets. (a) Original image. (b) DeepLabV3+. (c) PSPNet. (d) U-net proposed in this study.

To further verify that the U-net convolutional neural network proposed in this study has good performance for the segmentation of leukocytes, two different semantic segmentation models are used for comparative analysis on the Cellavision and BCCD public datasets.  Figure 9(a) shows two raw random images taken from these two datasets. DeepLabV3+ [17], PSPNet [18], and U-net [19] are the comparative methods. The comparison of segmentation results is shown in Figure 9. These approaches can yield relatively optimal segmentation results according to the comparison results. However, U-net has better results than the other two methods. In terms of edge processing, the other two techniques have flaws. For example, the edge processing of the DeepLabV3+ model is too sharp, removing details such as cytoplasm; the performance of the PSPNet models even makes leukocytes incomplete and loses the characteristics of leukocytes. The best performing method is the segmentation method suggested in this study, which overcomes the foregoing difficulties and produces fairly standard segmentation results.

Table 2 represents the parameter comparison of different methods for leukocyte segmentation. It can be seen that the U-net proposed in this study achieves the highest accuracy.

**Figure 10:**
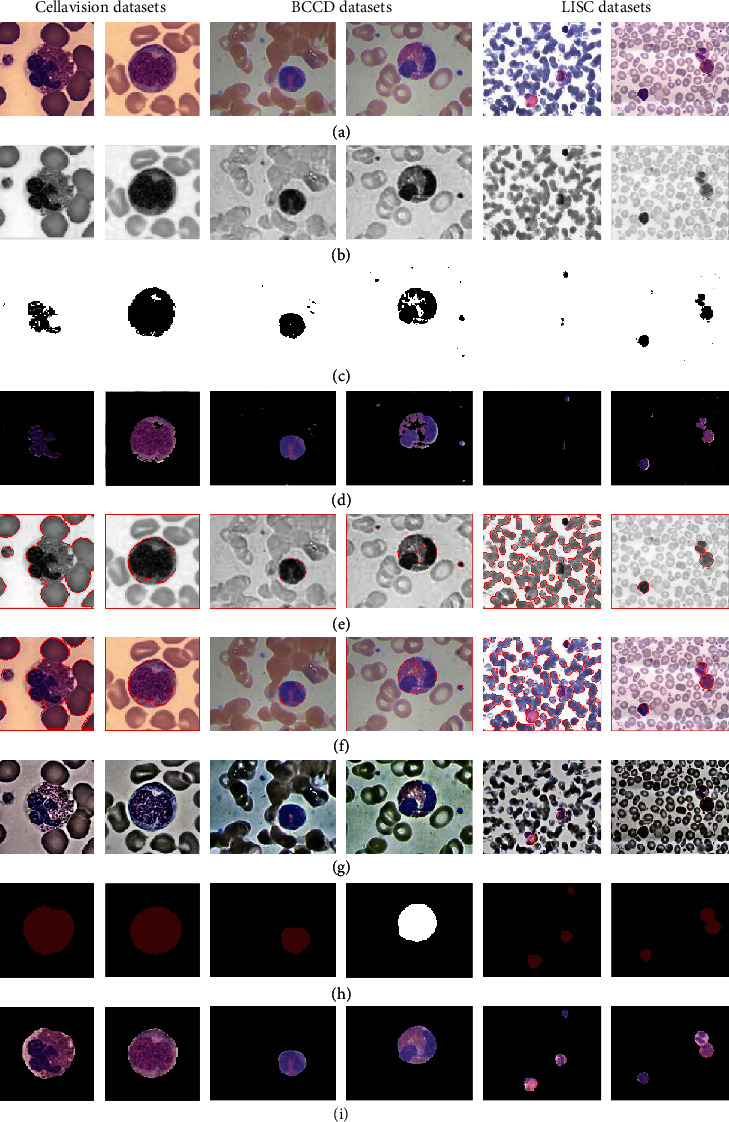
Segmentation results of different methods on leukocytes. (a) Original image. (b) RGB to CIE image. (c) Mask segmented by the method of [20] (Local Adaptive Threshold). (d) The segmentation result of the fusion of (a) and (c). (e) Segmented results by the method of [21] (Feedback-based Watershed Algorithm Aided Multiple Thresholds). (f) The segmentation result of the fusion of (a) and (e). (g) Adaptive retinex processing results. (h) Mask segmented by the method of this study (adaptive retinex correction and U-net). (i) The segmentation result of the fusion of (a) and (h).

In recent years, in addition to using CNNs for leukocyte segmentation, traditional segmentation methods have also been widely favour by researchers, as the locally adaptive threshold segmentation method mentioned [20] and the feedback-based watershed algorithm aided multiple thresholds [21]. Moreover, in [20, 22], an image preprocessing method converting RGB to CIE to solve the problem of uneven illumination of the collected cell images is used. In this subsection, the benefits and drawbacks of the preprocessing method proposed in this study and those proposed in [20, 22] will be discussed for comparison. At the same time, the results of the segmentation method proposed in this study and in [20, 21] in leukocyte segmentation will be discussed. The results are shown in Figure 10.

To solve the problems of similar colour and uneven illumination of other cells and leukocytes in blood cell images, two preprocessing methods were used to conduct comparative experiments on three different datasets. Line (a) in Figure 10 converts the original RGB colour space to CIE L∗u∗v∗. In fact, L∗u∗v∗ is an excellent intensity (represented by lightness L∗) and chromaticity (denoted by u∗ and v∗ components) decoupler [20]. The results of the adaptive retinex correction proposed in this study are shown in line (g). Comparing these two methods, it is found that the method in this study achieves better results for blood cell images. In the Cellavision and LISC datasets, the processed CIE images still have the problem that the colour of leukocytes is similar to that of other cells, and even in the LISC dataset, there is a fusion effect. In addition, this study also adopted three segmentation methods to segment leukocytes. Lines (c) and (d) are the method of [20], (c) is the mask obtained by using the CIE image to perform local adaptive threshold segmentation, and (d) is the segmentation result after fusion of the original blood cell image and the mask. Lines (e) and (f) are the method of [21], (e) is the result of the feedback-based watershed algorithm aided multiple threshold segmentation using the CIE image, and (f) is the segmentation result plotted on the original blood cell image. Lines (h) and (i) are the method proposed in this study, (h) is the segmentation result predicted by the U-net neural network on the image processed by adaptive retinex correction, and (i) is the leukocyte obtained by fusing the original blood cell image with the segmentation result. Comparing these three methods, in the Cellavision dataset, neither [20] nor [21] achieved satisfactory results. Because leukocytes are too similar to other cells, there is a problem of less segmentation of the cytoplasm. There is also such a problem in the BCCD dataset, and the additional question is to segment the impurities; there is even a loss of leukocytes in the LISC dataset. In addition to the problems listed above, the method of [21] showed that other cells were also segmented. However, the segmentation results presented by the method proposed in this study are good and meet the requirements of leukocyte segmentation.

### 4.3. Leukocyte Count

White blood cell count refers to counting the number of white blood cells contained in a unit volume of blood. Inflammation or other diseases in the body can cause changes in the total number of white blood cells, so counting the number of white blood cells is also worthy of attention. Through the discussion in Sections 4.1 and 4.2, the white blood cell segmentation method based on adaptive retinex correction and U-net proposed in this study has a good segmentation effect and can obtain well-segmented white blood cells with background removed and U-net predicted with red colour block-segmented image. Because the prediction result contains a certain number of red colour blocks, these red colour blocks represent the white blood cells in the image. Therefore, in this study, the number of white blood cells in the blood cell image was determined by determining the number of red colour blocks.

**Figure 11:**
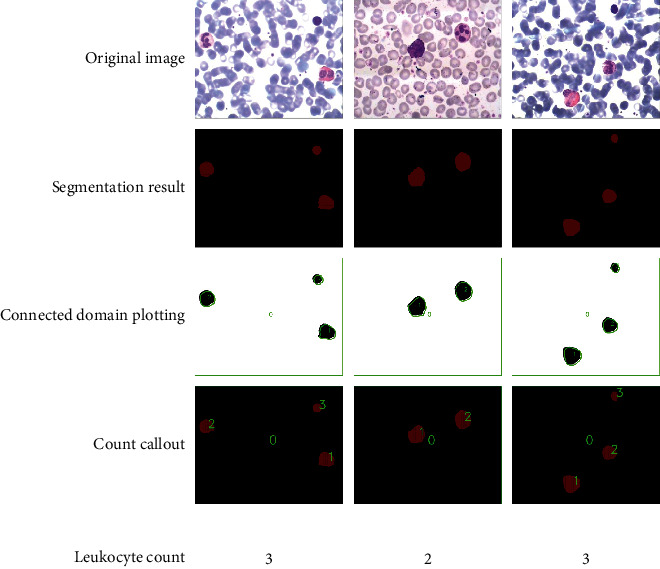
Leukocyte counts.

As shown in Figure 11, first, the segmentation result map is obtained by using the leukocyte segmentation method proposed in this study; then the image is binarized, and the connected domain is found and drawn; finally, the number of leukocytes is marked in order in the segmentation result graph, among which the black background is 0. This method preliminarily met the requirement of counting white blood cells.

## 5. Conclusions

This study proposes a leukocyte segmentation method based on adaptive retinex correction and U-net. First, the adaptive retinex method is used to process blood cell images taken under the microscope, increasing the clarity of leukocyte features and eliminating the problem of uneven lighting during acquisition, using the OpenCV platform. After that, leukocyte segmentation is performed on the processed image using the U-net convolutional neural network. The performance and accuracy of leukocyte segmentation are increased to some extent when compared to the unprocessed original blood cell image, and the number of data samples needed is lowered as well. MIoU grew by 22.09%, MPA grew by 15.53%, and accuracy grew by 15.24%.

Compared with the method of converting RGB to CIE, the adaptive retinex correction proposed in this study is more suitable for blood cell images, effectively distinguishing leukocytes from other cells and impurities, and enhancing the detailed characteristics of leukocytes, laying a foundation for subsequent segmentation. This study also compares the proposed U-net network with the DeepLabV3+ and PSPNet networks, achieving over 94% accuracy on both the Cellavision and BCCD datasets.

Compared with the method in [20, 21], the method proposed in this study has better performance on multiple datasets, and the performance of segmentation is even better. An automated method for leukocyte segmentation is provided.

To further increase the practicality of the method of this study, the count of leukocytes was added, and currently only independent leukocytes were counted. There are still great challenges for the counting of adherent or coincident cells, but it can be considered to use the proportion of the area occupied by leukocytes in the whole blood cell image to achieve the count, which can be extended to the problem of quantifying cells or colonies. This is also a future research direction of this study.

## Figures and Tables

**Table 1:** Comparison of parameters before and after processing by this method.

Sample	Number of data samples	Dice	MIoU (%)	MPA (%)	Accuracy (%)
Raw image dataset	80	0.771	70.83	82.3	83.63
Processed dataset	40	0.944	92.92	97.83	98.87

**Table 2:** Comparison of parameters of different methods under different datasets.

Datasets	Method	Dice	MIoU (%)	MPA (%)	Accuracy (%)
Cellavision datasets	DeepLabV3+	0.837	75.12	86.81	90.90
	PSPNet	0.828	74.69	86.06	89.49
	U-net	0.898	87.52	93.02	94.98

BCCD datasets	DeepLabV3+	0.804	76.48	85.19	88.33
	PSPNet	0.772	73.89	83.84	86.15
	U-net	0.901	88.75	92.55	94.27

## Data Availability

Previously reported [LISC] data were used to support this study and are available at http://users.cecs.anu.edu.au/~hrezatofighi/Data/Leukocyte%20Data.htm/. These prior studies (and datasets) are cited at relevant places within the text as references [15]. Previously reported [Cellavision] data were used to support this study and are available at https://data.mendeley.com/datasets/w7cvnmn4c5/1. These prior studies (and datasets) are cited at relevant places within the text as references [16]. Previously reported [BCCD] data were used to support this study and are available at https://public.roboflow.com/object-detection/bccd.
